# Knowledge, attitudes, and practices regarding vitamin D among adults in Palestine: a cross-sectional study

**DOI:** 10.3389/fpubh.2026.1868025

**Published:** 2026-07-14

**Authors:** Wadie Bashar Qaqunda, Elias Amarneh, Tareq Muallem, Ameer Sarhan, Mohammed Amouri

**Affiliations:** Faculty of Medicine, Al-Quds University, Jerusalem, Palestine

**Keywords:** attitude, knowledge, Palestine, practices, public health awareness, vitamin D deficiency.

## Abstract

**Background:**

Vitamin D deficiency is highly prevalent in the Middle East, including Palestine, despite abundant sunlight. Limited data exist on knowledge, attitudes, and practices (KAP) regarding vitamin D among the general Palestinian population.

**Methods:**

A cross-sectional, questionnaire-based study was conducted among 402 Palestinian adults between March and April 2026 using an online survey distributed via social media. The questionnaire assessed sociodemographic characteristics and vitamin D–related knowledge, attitudes, and practices. Knowledge was measured using a 16-item scale (score range 0–16). Statistical analyses included descriptive statistics, bivariate tests, and multivariable linear regression to identify independent predictors of knowledge.

**Results:**

The mean knowledge score was 8.15 ± 2.41 out of 16 (50.9%), and 68.4% of participants achieved at least half of the total score. Awareness of core concepts was high, but misconceptions persisted regarding nonspecific symptoms and overstated immune-related claims. Attitudes were generally favorable; however, although 76.9% of respondents recognized vitamin D deficiency as a societal health issue, only 49.0% perceived themselves to be personally at risk. Practices varied considerably: 38.1% reported daily sun exposure, and most participants exposed only limited body areas. In the final multivariable model, health-field affiliation, city residence, regular vitamin D supplementation, and non-smoking were independently associated with higher knowledge, whereas occasional supplementation was not.

**Conclusion:**

Palestinian adults demonstrate moderate but incomplete knowledge of vitamin D, with persistent misconceptions and a clear gap between awareness and practice. Targeted public health interventions that correct misinformation, improve health literacy, and promote evidence-based behaviors—particularly among village/camp residents and non-health professionals—are needed to improve vitamin D-related outcomes.

## Introduction

Vitamin D is a crucial fat-soluble secosteroid hormone essential for maintaining calcium and phosphate homeostasis, bone health, and overall physiological function (1). Beyond its well-established role in preventing rickets and osteomalacia, emerging evidence suggests a role for vitamin D in immune modulation and autoimmune diseases, with additional associations reported for cancer, although these relationships remain under investigation (2). Despite its critical importance, vitamin D deficiency (VDD) remains a widespread global public health problem, with estimates suggesting that up to one billion people may be affected worldwide (3). The Middle East and North Africa (MENA) region, including Palestine, exhibits a particularly high prevalence of VDD, often paradoxically so, given the abundant sunshine (4). This high prevalence is attributed to a complex interplay of factors, including cultural practices that limit sun exposure (e.g., traditional clothing), insufficient dietary intake of vitamin D-rich foods, genetic predispositions, and limited public awareness regarding optimal vitamin D sources and requirements (4, 5). In the Palestinian context, evidence points to widespread vitamin D deficiency and insufficient knowledge and practices among the population, with socioeconomic factors and complex political realities likely playing a role in shaping health-related behaviors. (6, 7). Studies from neighboring countries like Jordan, Saudi Arabia, and Lebanon have consistently reported high rates of VDD across various demographic groups, highlighting a regional challenge (8–10). A recent multi-Arab countries study also highlighted variable knowledge, attitudes, and practices related to vitamin D supplement use among adults, supporting the need for country-specific assessments of vitamin D awareness and behavior (11). For instance, research in Jordan has shown that VDD and insufficiency are significant public health problems among women, with prevalence rates as high as 95.7% in some cohorts, primarily linked to limited sun exposure and low dietary intake (12). In Palestine, specific data on the KAP regarding vitamin D among the general adult population are relatively scarce. However, existing research has highlighted the severity of the issue; for example, one study of 2,333 patients found that 78.7% were classified as having VDD (6). Another study of 400 Palestinian university students found that 57.8% possessed poor knowledge about the vitamin, while 57.5% exhibited negative health practices, and 53.8% were classified as “unaware” regarding the vitamin (7). A study of West Bank pharmacists revealed that while most hold positive attitudes and personally use dietary supplements for general wellbeing, their professional knowledge remains moderate (mean 3.68/5). Significant gaps persist, with only 3.6% understanding how to treat nutrient deficiencies. Moreover, 73.2% provide patient advice based on their own knowledge rather than specialist referrals (13). Given the alarming prevalence of VDD in Palestine and the identified knowledge gaps in specific subgroups, a thorough assessment of the broader adult population's knowledge, attitudes, and practices is imperative to inform public health strategies and educational campaigns tailored to the Palestinian context. This study aims to fill this knowledge gap by evaluating the KAP regarding vitamin D among adults in Palestine. By understanding the current levels of awareness, perceptions, and behaviors related to vitamin D, this research seeks to identify specific areas where educational interventions are most needed. The findings will contribute to the development of evidence-based public health initiatives designed to improve vitamin D status and overall health outcomes within the Palestinian community.

## Methodology

### Study design and setting

This study was designed as a cross-sectional, questionnaire-based survey to assess knowledge, attitudes, and practices regarding vitamin D among adults in Palestine. Data were collected between 24 March 2026 and 8 April 2026 using a structured, self-administered online questionnaire created on Google Forms. The survey link was distributed electronically through commonly used social media platforms and personal networks, including WhatsApp, Facebook, and similar channels, using a convenience snowball approach to maximize reach across different Palestinian communities. This approach allowed rapid dissemination and broad participation; however, as with other online community surveys, it may have favored younger, more educated, and more digitally connected individuals.

### Participants

The target population consisted of Palestinian adults aged 18 years and older who were able to read and respond to the questionnaire electronically. Both males and females from different areas of residence were eligible to participate, including respondents living in cities, villages, and camps, as well as individuals from different geopolitical areas connected to Palestine. Participants were included if they completed the online survey and provided consent electronically before submission. Responses that were incomplete or did not meet the study eligibility criteria were excluded from the final analysis. A total of 402 complete responses were included in the final dataset.

### Questionnaire development and translation

The questionnaire was developed after reviewing multiple previously published studies that used valid and reliable instruments to assess vitamin D-related knowledge, attitudes, and practices. Items were selected from these published questionnaires and then supplemented with additional questions based on the scientific background, clinical experience, and research interests of the study team to ensure that the final tool adequately addressed issues relevant to the Palestinian context. After drafting the questionnaire, it was reviewed by subject experts to evaluate content relevance, clarity, wording, and cultural suitability. Their comments were incorporated before final administration, and the resulting questionnaire was considered suitable for use in the target population.

After the questionnaire was finalized, it was translated into Arabic and administered online in Arabic to facilitate comprehension and ensure that participants could respond in their native language. Following completion of data collection, the questionnaire content, variable labels, and response categories were translated into English for data coding, statistical analysis, and manuscript preparation. This process was carried out carefully to preserve the original meaning of the questions and the conceptual consistency of the collected responses across both languages.

The final questionnaire consisted of several sections. These included sociodemographic characteristics, health-related background information, knowledge items related to vitamin D, attitude and perception statements, and practice-related questions addressing sun exposure, vitamin D testing, and supplementation behavior. The instrument was designed to provide a comprehensive evaluation of public understanding and behavior regarding vitamin D in the Palestinian adult population.

### Scoring and reliability

Knowledge regarding vitamin D was assessed using a 16-item scale covering major domains such as the physiological role of vitamin D, sources of vitamin D, consequences of deficiency, common misconceptions, and methods of testing. Each item had three response options: “Yes,” “No,” and “I don't know.” Responses were coded as 1 for a correct answer and 0 for an incorrect or uncertain answer. The total knowledge score therefore ranged from 0 to 16, with higher scores indicating better knowledge. For descriptive and comparative analysis, participants were also classified according to whether they achieved at least half of the total obtainable score.

Attitudes toward vitamin D were assessed using a series of Likert-type statements addressing perceived importance of vitamin D, perceived risk of deficiency, views on supplementation, and beliefs about testing and sun exposure. Practice items assessed self-reported behaviors such as duration and frequency of sun exposure, intentional exposure habits, vitamin D testing, supplement use, dosage awareness, and source of recommendation. Composite scores were generated where appropriate for the main analytical domains, while selected practice variables were also analyzed individually according to the objectives of the study.

The internal consistency of the study instrument was evaluated using Cronbach's alpha. All scales demonstrated good internal reliability, with Cronbach's alpha values greater than 0.79, indicating acceptable consistency among the items used to measure the principal study constructs.

### Data management and statistical analysis

Data were exported from Google Forms into Microsoft Excel and then imported into IBM SPSS Statistics version 25.0 (IBM Corp., Armonk, NY, USA) for cleaning and statistical analysis. Because the survey was completed in Arabic, the dataset and variable labels were translated into English before formal coding and analysis. Data cleaning included checking for completeness, consistency of coding, duplication, and logical compatibility between related variables. Variables were then recoded as needed for descriptive, comparative, and regression analyses.

Descriptive statistics were used to summarize participant characteristics and the main study variables. Categorical variables were presented as frequencies and percentages, whereas continuous variables were summarized as means and standard deviations. Associations between categorical variables were examined using the chi-square test. Comparisons of mean knowledge scores between two groups were performed using Welch's *t*-test, while comparisons across more than two groups were conducted using one-way analysis of variance (ANOVA). For significant ANOVA results, Tukey's honestly significant difference test was used for *post-hoc* pairwise comparisons. Multivariable linear regression was used to identify independent predictors of knowledge score. Regression assumptions were assessed by examining residuals, influential observations, and multicollinearity; variance inflation factor (VIF) and tolerance were calculated for all predictors. Because diagnostic assessment suggested mild heteroskedasticity, the final regression model is reported using HC3 robust standard errors, 95% confidence intervals, and two-sided *p*-values. Statistical significance was set at *p* < 0.05.

## Ethical approval

Ethical review and approval were waived for this study by the Research Ethics Committee of Al-Quds University, Jerusalem, Palestine, because the project involved an anonymous, minimal-risk online survey and did not collect direct personal identifiers. Participation was entirely voluntary. Before accessing the questionnaire, participants were presented with an electronic information and consent statement describing the study purpose, the voluntary nature of participation, confidentiality protections, and their right to discontinue participation before submitting the survey. Electronic informed consent was obtained from all participants before questionnaire access. The study was conducted in accordance with the Declaration of Helsinki (14).

## Results

A total of 402 adults from Palestine completed the questionnaire (Table 1). The study population was predominantly young, with 221 participants (55.0%) aged 18–25 years, while 73 (18.2%) were aged 26–35 years, 31 (7.7%) were aged 36–45 years, 63 (15.7%) were aged 46–60 years, and 14 (3.5%) were older than 60 years. Women accounted for 56.2% of respondents and men for 43.8%. Most participants resided in the West Bank (84.6%), with smaller proportions from Jerusalem (10.4%) and other locations. Urban residence was common, as 296 participants (73.6%) lived in cities and 106 (26.4%) lived in villages or camps. Educational attainment was generally high, with nearly two-thirds holding a bachelor's degree. In terms of occupation, 38.1% were employed and 32.8% were students. A health-related academic or professional background was reported by 19.2% of respondents. Most participants did not report a chronic disease (85.8%), and 31.6% were current smokers.

**Table 1:** Sociodemographic characteristics of the study sample (*N* = 402).

Variable	Category	*n* (%)
Age group	18–25 years	221 (55.0%)
	26–35 years	73 (18.2%)
	36–45 years	31 (7.7%)
	46–60 years	63 (15.7%)
	Over 60 years	14 (3.5%)
Sex	Male	176 (43.8%)
	Female	226 (56.2%)
Geopolitical residence	West Bank	340 (84.6%)
	Jerusalem	42 (10.4%)
	Palestinian living outside Palestine	16 (4.0%)
	Israel	2 (0.5%)
	Gaza Strip	2 (0.5%)
Locality	City	296 (73.6%)
	Village	99 (24.6%)
	Camp	7 (1.7%)
Governorate	Ramallah and Al-Bireh	266 (66.2%)
	Bethlehem	32 (8.0%)
	Jerusalem	28 (7.0%)
	Hebron	21 (5.2%)
	Jericho	11 (2.7%)
	Other governorates	44 (10.9%)
Educational level	Primary	10 (2.5%)
	Secondary (high school)	43 (10.7%)
	Diploma/Professional	28 (7.0%)
	Bachelor's	260 (64.7%)
	Postgraduate studies (Master's/PhD)	61 (15.2%)
Job status	Employee (government/private)	153 (38.1%)
	Student	132 (32.8%)
	Not currently working	61 (15.2%)
	Freelance work	47 (11.7%)
	Retired	9 (2.2%)
Health-field status	Yes	77 (19.2%)
	No	325 (80.8%)
Monthly income	Unemployed/student	147 (36.6%)
	<2,000 NIS	18 (4.5%)
	2,000–5,000 NIS	94 (23.4%)
	>5,000 NIS	67 (16.7%)
	Prefer not to answer	76 (18.9%)
Marital status	Single	254 (63.2%)
	Married	142 (35.3%)
	Divorced	4 (1.0%)
	Widower	2 (0.5%)
Current smoking	Yes	127 (31.6%)
	No	275 (68.4%)
Chronic disease	Yes	57 (14.2%)
	No	345 (85.8%)

The mean total knowledge score was 8.15 ± 2.41 out of 16, equivalent to 50.9% of the maximum attainable score. Overall, 275 participants (68.4%) answered at least half of the knowledge items correctly, whereas 107 (26.6%) achieved a score of 10 or higher. Clear variation was observed across several participant characteristics (Table 2). Women had higher mean scores than men (8.39 ± 2.06 vs. 7.84 ± 2.78, *p* = 0.026). Participants living in cities also scored higher than those living in villages or camps (8.35 ± 2.41 vs. 7.58 ± 2.33, *p* = 0.004). The largest difference was seen according to health-field affiliation, as respondents working or studying in a health-related field had markedly higher scores than others (9.10 ± 1.95 vs. 7.92 ± 2.46, *p* < 0.001). Non-smokers likewise had higher scores than smokers (8.37 ± 2.35 vs. 7.68 ± 2.48, *p* = 0.009). By contrast, no significant differences in total knowledge score were observed according to age group, geopolitical residence, educational level, job status, monthly income, or chronic disease status (all *p* > 0.05).

**Table 2:** Knowledge score comparisons according to sociodemographic and behavioral characteristics.

Variable	Category	*n*	Score (mean ±SD)	Test	Effect size	*p*
Sex	Male	176	7.84 ± 2.78	*t* = -2.23	*d* = 0.23	0.026
	Female	226	8.39 ± 2.06			
Age group	18–25 years	221	8.00 ± 2.48	*F* = 0.68	η^2^ = 0.01	0.607
	26–35 years	73	8.18 ± 2.16			
	36–45 years	31	8.29 ± 2.34			
	46–60 years	63	8.52 ± 2.30			
	Over 60 years	14	8.43 ± 3.23			
Geopolitical residence	West Bank	340	8.06 ± 2.34	*F* = 1.36	η^2^ = 0.01	0.257
	Jerusalem	42	8.60 ± 3.04			
	Other	20	8.65 ± 1.98			
Locality	City	296	8.35 ± 2.41	*t* = 2.88	*d* = 0.32	0.004
	Village/Camp	106	7.58 ± 2.33			
Educational level	Bachelor's	260	8.13 ± 2.35	*F* = 0.68	η^2^ = 0.01	0.608
	Postgraduate	61	8.31 ± 2.64			
	Secondary	43	7.93 ± 2.52			
	Diploma/Professional	28	7.96 ± 1.99			
	Primary	10	9.20 ± 3.19			
Monthly income	Unemployed/student	147	8.01 ± 2.62	*F* = 0.62	η^2^ = 0.01	0.647
	<2,000 NIS	18	8.50 ± 2.81			
	2,000–5,000 NIS	94	7.98 ± 2.32			
	>5,000 NIS	67	8.36 ± 2.29			
	Prefer not to answer	76	8.37 ± 2.10			
Health-field status	No	325	7.92 ± 2.46	t = −4.53	*d* = 0.50	<0.001
	Yes	77	9.10 ± 1.95			
Current smoking	No	275	8.37 ± 2.35	*t* = 2.64	*d* = 0.29	0.009
	Yes	127	7.68 ± 2.48			
Chronic disease	No	345	8.09 ± 2.44	*t* = −1.24	*d* = 0.17	0.220
	Yes	57	8.49 ± 2.22			
Vitamin D supplementation	No	169	7.64 ± 2.61	*F* = 11.62	η^2^ = 0.06	<0.001
	Occasional	106	8.00 ± 2.22			
	Regular	127	8.95 ± 2.08			

For vitamin D supplementation, different superscript letters indicate statistically significant pairwise differences according to Tukey HSD post-hoc testing. Regular users had significantly higher knowledge scores than non-users and occasional users, whereas occasional users did not differ significantly from non-users.

**Figure 1:**
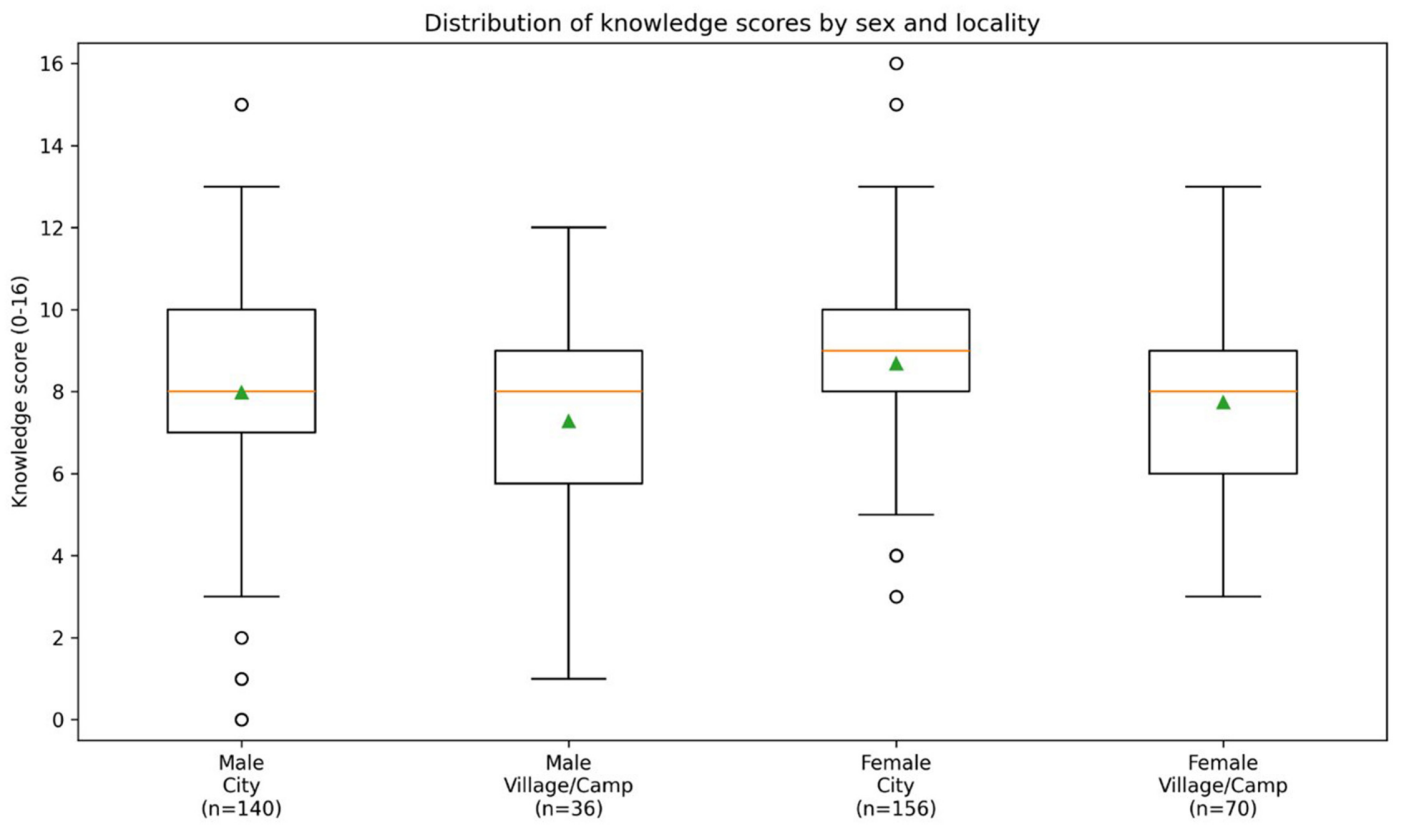
Distribution of total knowledge scores by sex and locality. Boxes represent the interquartile range, center lines indicate the median, and means are shown by the default boxplot marker.

Figure 1 illustrates the distribution of knowledge scores according to sex and locality. The pattern remained consistent across groups, with women generally scoring higher than men and city residents generally scoring higher than village or camp residents. When these two factors were considered together, both sex and locality retained independent associations with knowledge score (*p* = 0.007 and *p* = 0.002, respectively), whereas there was no evidence that the sex difference varied according to locality (*p* = 0.665). Thus, the advantage observed among women and among urban residents appeared to reflect parallel rather than interacting effects.

**Figure 2:**
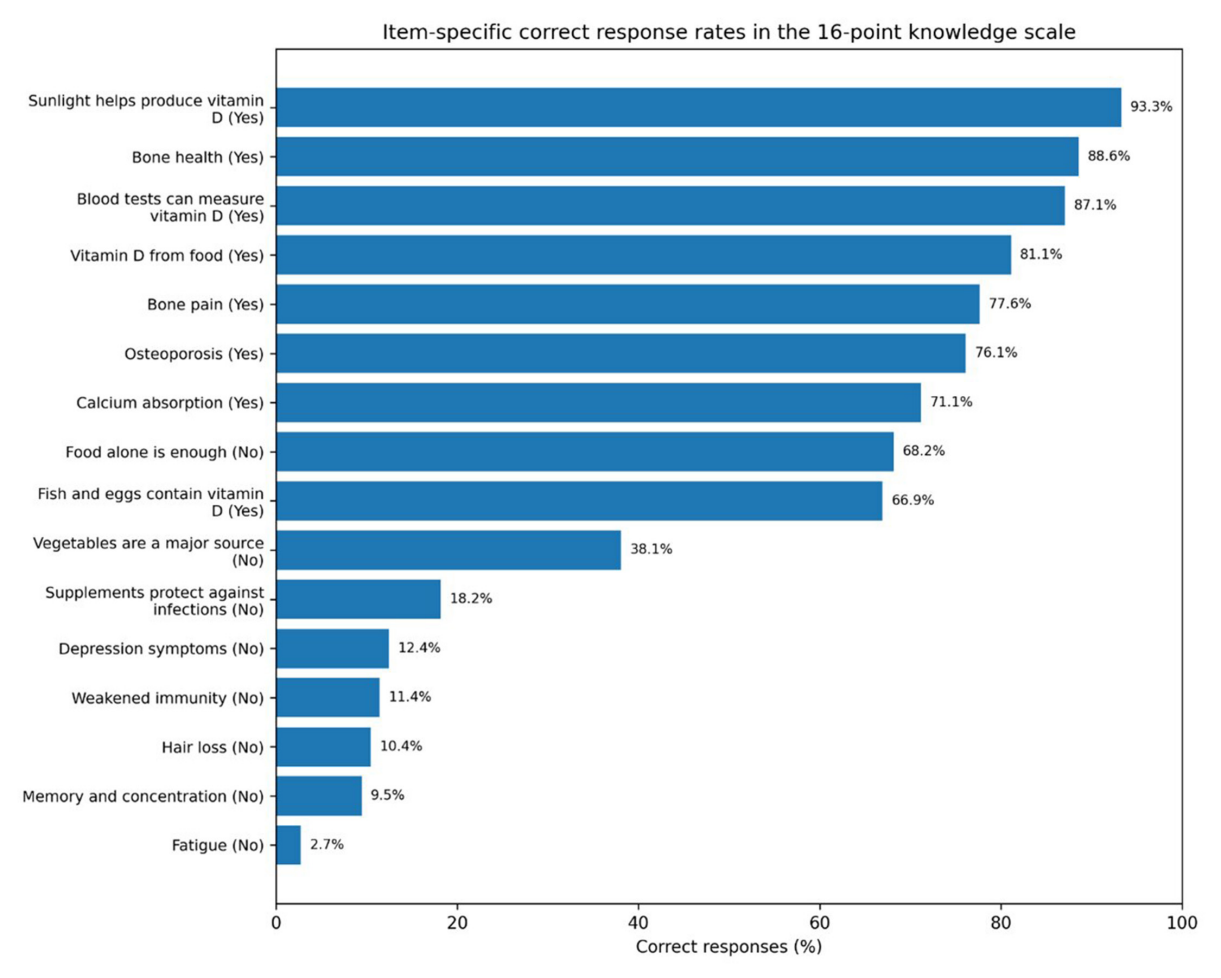
Item-specific correct response rates for the 16 scored vitamin D knowledge items. For positively keyed statements, the correct response was “Yes”; for negatively keyed statements, the correct response was “No.”

At the item level, participants demonstrated stronger knowledge of established vitamin D concepts than of nonspecific or overstated claims (Figure 2). Correct response rates were highest for recognizing sunlight as a source of vitamin D synthesis (93.3%), acknowledging the importance of vitamin D for bone health (88.6%), identifying blood testing as a way to assess vitamin D status (87.1%), and recognizing food as a source of vitamin D (81.1%). Performance was also relatively strong for bone pain (77.6%), osteoporosis (76.1%), calcium absorption (71.1%), and correctly rejecting the statement that diet alone is sufficient for most people (68.2%). In contrast, misconceptions were common for several negatively keyed items. Only 2.7% correctly rejected fatigue, 9.5% correctly rejected memory and concentration problems, 10.4% correctly rejected hair loss, 11.4% correctly rejected weakened immunity, and 12.4% correctly rejected depression symptoms as typical manifestations. Likewise, only 18.2% correctly rejected the statement that vitamin D supplements protect against bacterial or viral infections, and only 38.1% correctly identified the statement that vegetables are a major source of vitamin D as false. Overall, the item-level pattern indicates reasonable knowledge of core concepts but substantial uncertainty regarding symptom-based and popularly circulated claims.

Attitudes toward vitamin D were generally favorable, although several responses indicated continuing uncertainty (Table 3). Most respondents agreed that vitamin D deficiency is a common health problem in society (76.9%), that regular sun exposure is important for health (78.6%), and that regular testing of vitamin D levels is essential (64.7%). Fewer than half believed themselves to be personally at risk of deficiency (49.0%), and 18.2% agreed that they rely on social media as a primary source of health information. Reported practices also showed marked variation. The most common sun-exposed body areas were the face and hands only (43.5%) and the face, hands, and arms (42.3%), whereas 12.4% reported exposing larger body areas. Daily sun exposure was reported by 38.1% of participants, and the most common exposure duration was 10–30 min per day. When asked how they would respond to symptoms such as fatigue, pain, or hair loss, 56.7% reported that they would visit a doctor, whereas the remainder reported self-management or other non-medical responses.

**Table 3:** Selected knowledge, attitude, and practice indicators according to sex and locality.

Indicator	Overall n/N (%)	Male n/N (%)	Female n/N (%)	Sex *χ^2^*	*p*	City n/N (%)	Village/camp n/N (%)	Area *χ^2^*	*p*
Adequate knowledge (Score ≥8)	275/402 (68.4%)	112/176 (63.6%)	163/226 (72.1%)	2.92	0.088	210/296 (70.9%)	65/106 (61.3%)	2.92	0.088
Deficiency common in society (agree)	309/402 (76.9%)	115/176 (65.3%)	194/226 (85.8%)	22.24	<0.001	229/296 (77.4%)	80/106 (75.5%)	0.07	0.793
Perceived personal risk (agree)	197/402 (49.0%)	70/176 (39.8%)	127/226 (56.2%)	10.03	0.002	140/296 (47.3%)	57/106 (53.8%)	1.06	0.302
Supplements safe without medical advice (agree)	88/402 (21.9%)	48/176 (27.3%)	40/226 (17.7%)	4.76	0.029	56/296 (18.9%)	32/106 (30.2%)	5.16	0.023
Social media primary source (agree)	73/402 (18.2%)	41/176 (23.3%)	32/226 (14.2%)	4.96	0.026	43/296 (14.5%)	30/106 (28.3%)	9.06	0.003
Regular vitamin D testing essential (agree)	260/402 (64.7%)	103/176 (58.5%)	157/226 (69.5%)	4.72	0.030	197/296 (66.6%)	63/106 (59.4%)	1.43	0.231
Daily sun exposure	153/402 (38.1%)	100/176 (56.8%)	53/226 (23.5%)	45.32	<0.001	108/296 (36.5%)	45/106 (42.5%)	0.94	0.333
Midday sun exposure	229/402 (57.0%)	98/176 (55.7%)	131/226 (58.0%)	0.13	0.721	179/296 (60.5%)	50/106 (47.2%)	5.10	0.024
Any vitamin D supplementation	233/402 (58.0%)	85/176 (48.3%)	148/226 (65.5%)	11.31	<0.001	178/296 (60.1%)	55/106 (51.9%)	1.85	0.173
Regular vitamin D supplementation	127/402 (31.6%)	42/176 (23.9%)	85/226 (37.6%)	8.03	0.005	96/296 (32.4%)	31/106 (29.2%)	0.23	0.628
Checked vitamin D before supplements	184/352 (52.3%)	65/152 (42.8%)	119/200 (59.5%)	9.04	0.003	143/257 (55.6%)	41/95 (43.2%)	3.85	0.050
Vitamin D test during past year	174/360 (48.3%)	62/156 (39.7%)	112/204 (54.9%)	7.54	0.006	133/262 (50.8%)	41/98 (41.8%)	1.93	0.164
Uses high-dose regimen	131/340 (38.5%)	33/145 (22.8%)	98/195 (50.3%)	25.40	<0.001	92/245 (37.6%)	39/95 (41.1%)	0.22	0.638
Would visit doctor if symptomatic	228/402 (56.7%)	100/176 (56.8%)	128/226 (56.6%)	0.00	1.000	172/296 (58.1%)	56/106 (52.8%)	0.68	0.408
Doctor-recommended supplementation	176/307 (57.3%)	55/122 (45.1%)	121/185 (65.4%)	11.60	<0.001	128/224 (57.1%)	48/83 (57.8%)	0.00	1.000

Vitamin D supplementation and testing behaviors were common but not universal. During the previous 12 months, 233 participants (58.0%) reported taking vitamin D supplements, including 127 (31.6%) who used them regularly and 106 (26.4%) who used them occasionally, while 169 (42.0%) reported no supplementation. Mean knowledge scores differed significantly across these three groups (Table 2): 8.95 ± 2.08 among regular users, 8.00 ± 2.22 among occasional users, and 7.64 ± 2.61 among non-users (one-way ANOVA, p <0.001). Tukey *post-hoc* comparisons showed that regular users had significantly higher scores than non-users (*p* < 0.001) and occasional users (*p* = 0.006), whereas occasional users did not differ significantly from non-users (*p* = 0.430). Among respondents with available follow-up data, 52.3% reported checking their vitamin D level before starting supplementation, 48.3% had undergone vitamin D testing during the previous year, 57.0% knew the dosage they were taking, and 38.5% reported using a high-dose weekly or monthly regimen. Doctors were the most commonly reported source of supplementation advice.

Several clinically relevant differences were observed according to sex and area of residence (Table 3). Women had greater odds than men of agreeing that vitamin D deficiency is a common societal health problem [odds ratio (OR) 3.22, 95% CI 1.98–5.23, *p* < 0.001] and of agreeing that regular vitamin D testing is essential (OR 1.61, 95% CI 1.07–2.44, *p* = 0.023). Women were also more likely to report regular vitamin D supplementation during the previous year (OR 1.92, 95% CI 1.24–2.98, *p* = 0.003), to report checking their vitamin D level before starting supplementation (OR 1.97, 95% CI 1.28–3.02, *p* = 0.002), and to know the supplement dosage they were taking (OR 2.47, 95% CI 1.57–3.87, *p* < 0.001). In addition, women had substantially greater odds of reporting a high-dose weekly or monthly supplementation regimen (OR 3.43, 95% CI 2.12–5.54, *p* < 0.001). In contrast, men were more likely to report sun exposure at least 3 times per week (OR 3.82, 95% CI 2.33–6.26, *p* < 0.001). Area-based contrasts were fewer but still informative. Compared with city residents, participants living in villages or camps had higher odds of identifying social media as their primary health information source (OR 2.32, 95% CI 1.36–3.95, *p* = 0.002), whereas city residents had higher odds of reporting that they checked their vitamin D level before starting supplementation (OR 1.65, 95% CI 1.03–2.66, *p* = 0.037). Most other sex- or area-specific differences were small and not statistically significant.

**Figure 3:**
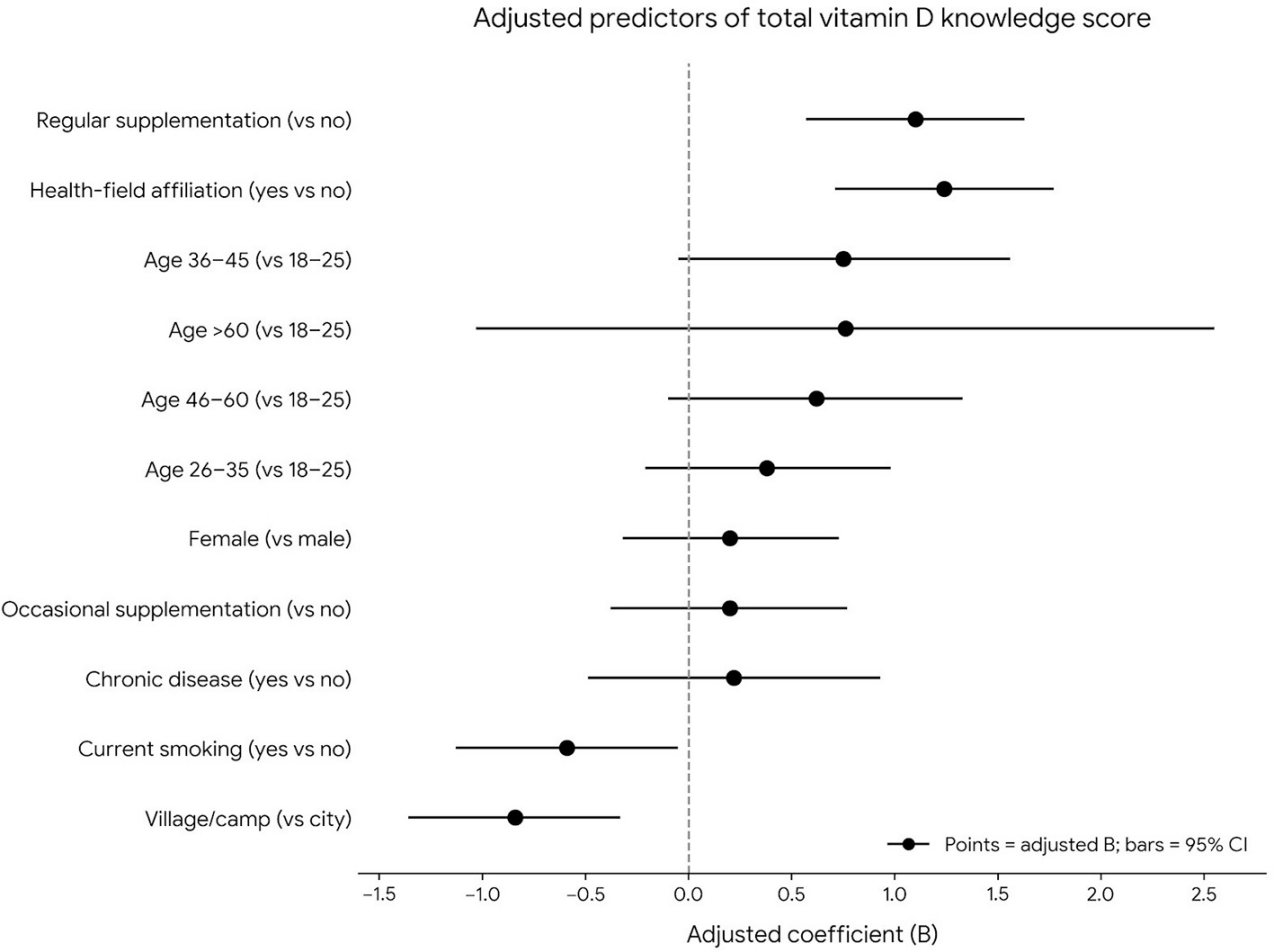
Adjusted regression coefficients for predictors of total knowledge score in the final multivariable linear regression model. Points indicate unstandardized coefficients and horizontal lines indicate 95% confidence intervals estimated with HC3 robust standard errors; the dashed vertical line marks the null value. Reference categories were male sex, age 18–25 years, city residence, no health-field affiliation, non-smoking, no chronic disease, and no vitamin D supplementation.

In the final multivariable linear regression model (Figure 3; Table 4), health-field affiliation was independently associated with a higher knowledge score (*B* = 1.24, SE = 0.27, 95% CI 0.71–1.77, *p* < 0.001), and regular vitamin D supplementation was also associated with a higher score compared with no supplementation (*B* = 1.10, SE = 0.27, 95% CI 0.57–1.63, *p* < 0.001). Compared with city residents, village/camp residents had lower adjusted knowledge scores (*B* = −0.84, SE = 0.26, 95% CI −1.36 to −0.33, *p* = 0.001), and current smoking was associated with lower knowledge (*B* = −0.59, SE = 0.27, 95% CI −1.13 to −0.05, *p* = 0.032). Occasional supplementation was not independently associated with knowledge (*B* = 0.20, SE = 0.29, 95% CI −0.38 to 0.77, *p* = 0.503). Female sex, age group, and chronic disease were not independently associated with knowledge after adjustment. Multicollinearity was not problematic (VIF range 1.05–1.39; tolerance range 0.72–0.95). Residual diagnostics showed mild heteroskedasticity without evidence of a single unduly influential case; therefore, the final model is reported with robust standard errors.

**Table 4:** Multivariable linear regression model for total knowledge score.

Predictor	*B*	SE	95% CI	*p*
Female (vs. male)	0.20	0.27	−0.32 to 0.73	0.449
Age 26–35 years (vs. 18–25)	0.38	0.30	−0.21 to 0.98	0.209
Age 36–45 years (vs. 18–25)	0.75	0.41	−0.05 to 1.56	0.067
Age 46–60 years (vs. 18–25)	0.62	0.36	−0.10 to 1.33	0.090
Age >60 years (vs. 18–25)	0.76	0.91	−1.03 to 2.55	0.403
Village/camp (vs. city)	−0.84	0.26	−1.36 to −0.33	0.001
Health-field affiliation (yes vs. no)	1.24	0.27	0.71 to 1.77	<0.001
Current smoking (yes vs. no)	−0.59	0.27	−1.13 to −0.05	0.032
Chronic disease (yes vs. no)	0.22	0.36	−0.49 to 0.93	0.540
Occasional supplementation (vs. no)	0.20	0.29	−0.38 to 0.77	0.503
Regular supplementation (vs. no)	1.10	0.27	0.57 to 1.63	<0.001

## Discussion

This cross-sectional study provides a detailed assessment of the knowledge, attitudes, and practices (KAP) regarding vitamin D among 402 Palestinian adults. The findings reveal a moderate level of vitamin D knowledge (mean score: 8.15 ± 2.41), generally positive attitudes, but varied practices, with significant demographic disparities. These results underscore the need for targeted public health interventions to improve vitamin D status in the Palestinian population.

Our study found a mean knowledge score of 8.15 out of 16 (50.9%), indicating moderate but incomplete knowledge regarding vitamin D among Palestinian adults. Awareness of core concepts was strong for sunlight (93.3%), bone health (88.6%), blood testing (87.1%), and food as a vitamin D source (81.1%). However, misconceptions were prominent for several symptom-related and overstated claims. The lowest correct-response rates were observed for rejecting fatigue (2.7%), memory and concentration problems (9.5%), hair loss (10.4%), weakened immunity (11.4%), and depression symptoms (12.4%) as typical manifestations, as well as for rejecting the claim that vitamin D supplementation protects against bacterial and viral infections (18.2%). In addition, only 38.1% correctly rejected vegetables as a major source of vitamin D. These findings suggest that broadly known concepts are more familiar than symptom-based or popularly circulated claims, underscoring the need for public health messaging that clearly distinguishes established evidence from overgeneralization and health misinformation (15).

Attitudes toward vitamin D were generally favorable, yet a “perception-practice gap” was evident. While 76.9% of respondents agreed that vitamin D deficiency is a common health problem in society, only 49.0% perceived themselves to be personally at risk. This low personal risk appraisal is a critical barrier to preventive behavior (16). Interestingly, women were significantly more likely than men to view deficiency as a common societal problem (85.8% vs. 65.3%, *p* < 0.001) and to believe regular testing is essential (69.5% vs. 58.5%, *p* = 0.030).

Reliance on social media as a primary health information source (18.2%) was significantly higher among village and camp residents compared to city dwellers (28.3% vs. 14.5%, p = 0.003), suggesting a potential vulnerability to misinformation in rural populations. This finding may indicate increased susceptibility in these groups, consistent with studies emphasizing the importance of identifying populations at higher risk of exposure to health misinformation (15). This disparity in information sources could explain the lower knowledge scores observed in rural populations (7.58 vs. 8.35, *p* = 0.004).

This study revealed marked variability in vitamin D–related practices, particularly by sex. Although 78.6% of participants recognized the importance of sun exposure, only 38.1% reported daily exposure, and most limited exposure to small body areas (face and hands: 43.5%; face, hands, and arms: 42.3%), suggesting suboptimal conditions for vitamin D synthesis. Significant sex-based differences were observed. Men were more likely than women to report daily sun exposure (56.8% vs. 23.5%, *p* < 0.001) and had nearly 4 times higher odds of regular exposure, defined as sun exposure at least 3 times per week (OR = 3.82, 95% CI 2.33–6.26, *p* < 0.001). Body area exposure also differed significantly (*p* < 0.001), with women more likely to report limited exposure (57.5% exposing face and hands vs. 25.6% of men), while men more frequently exposed larger areas (57.4% face, hands, and arms vs. 30.5% of women; 15.3% vs. 10.2% for larger body areas). When grouped, women had significantly higher odds of limited exposure (OR = 3.88, 95% CI 2.54–5.94). Despite similar midday exposure patterns (55.7% vs. 58.0%, *p* = 0.721), men achieved greater overall sun exposure due to both higher frequency and broader skin exposure, indicating more effective cumulative UV exposure. In contrast, women demonstrated higher knowledge scores (8.39 vs. 7.84, *p* = 0.026) and greater engagement in preventive behaviors, including supplementation (37.6% vs. 23.9%, *p* = 0.005), high-dose use (50.3% vs. 22.8%, *p* < 0.001), and prior testing (59.5% vs. 42.8%, *p* = 0.003). However, sex was not an independent predictor of knowledge in the final adjusted model (*p* = 0.449), suggesting that this difference is better explained by higher engagement in health-related behaviors among women, as well as associated factors such as regular supplement use (*p* < 0.001), health-field affiliation (*p* < 0.001), and urban residence (*p* = 0.001), all of which were independently associated with higher knowledge scores. Overall, men exhibited higher sun exposure, whereas women demonstrated greater engagement in preventive health behaviors, highlighting a divergence between exposure-based and knowledge-driven practices.

The final multivariable model clarified the independent predictors of knowledge. Health-field affiliation remained the strongest positive predictor, which is consistent with the expected effect of formal health-related training. Regular vitamin D supplementation, but not occasional supplementation, was independently associated with higher knowledge, suggesting that sustained engagement with vitamin D-related health behavior may reflect or reinforce better health literacy. Compared with city residents, village/camp residents had lower adjusted knowledge scores, which is fully consistent with the higher mean score observed among city residents in the unadjusted analysis and should be interpreted as a reference-category comparison rather than a paradox. Current smoking also remained negatively associated with knowledge, highlighting smokers as a subgroup that may benefit from targeted health communication. Collectively, these findings identify village/camp residents, non-health professionals, and smokers as priority groups for future educational interventions.

The findings highlight important public health implications regarding vitamin D awareness, misconceptions, and health information behaviors in Palestine. The high prevalence of misconceptions about non-specific symptoms, along with the widespread reliance on social media as a health information source, underscores the need for targeted health education interventions. Public health campaigns delivered through social media, television, and primary healthcare settings should focus on correcting common myths and emphasizing evidence-based information, particularly the established roles of vitamin D in bone and immune health. In addition, tailored interventions such as rural outreach programs, mobile clinics, women's health and antenatal counseling, and physician training may improve knowledge and promote consistent guidance on testing and supplementation. Strengthening the role of trusted healthcare professionals and leveraging official digital platforms could further enhance the dissemination of accurate information. Promoting awareness of the simplicity and accessibility of vitamin D blood testing may also help translate knowledge into preventive health behaviors.

This study has several limitations that should be considered when interpreting the findings. The cross-sectional design limits the ability to infer causal relationships between knowledge, attitudes, and practices. In addition, the use of a convenience snowball sampling approach via online platforms may have introduced selection bias, potentially over representing younger, more educated, urban, and digitally connected individuals, which may limit the generalizability of the results to the broader Palestinian adult population. The reliance on self-reported data may also introduce recall bias and social desirability bias, particularly for behavioral practices such as supplementation and sun exposure. Furthermore, the study did not include objective biochemical measurements of serum vitamin D levels, which would have provided a more accurate assessment of actual deficiency status. Future research should consider longitudinal designs, probability-based sampling methods, and the inclusion of biochemical markers to provide a more comprehensive understanding of vitamin D status and related behaviors.

## Conclusion

This study demonstrates that Palestinian adults possess a moderate but incomplete understanding of vitamin D, reflected by a mean knowledge score of 8.15 ± 2.41 and the presence of a clear perception–practice gap. Although most participants recognized vitamin D deficiency as a common public health issue, fewer perceived themselves to be personally at risk, which may limit engagement in preventive behaviors. Notable disparities were observed across subgroups: women demonstrated higher knowledge levels and greater engagement in supplementation and testing practices, while individuals residing in urban areas exhibited higher knowledge levels. In contrast, men reported more frequent sun exposure but lower involvement in preventive health behaviors.

Multivariable analysis identified health-field affiliation, city residence, regular vitamin D supplementation, and non-smoking as independent correlates of higher knowledge. In addition, the persistence of misconceptions regarding non-specific symptoms, such as fatigue and hair loss, underscores the need for more precise and evidence-based health communication.

Improving vitamin D–related health outcomes in Palestine requires multifaceted public health strategies that address both knowledge gaps and behavioral patterns. Interventions should focus on enhancing health literacy, correcting common misconceptions, and improving access to reliable health information, particularly among rural populations and non-health professionals (17). Promoting appropriate sun exposure, evidence-based supplementation, and increased uptake of vitamin D testing may help translate awareness into effective preventive practices. Future research should incorporate objective biochemical assessments and longitudinal designs to better understand vitamin D status and evaluate the long-term impact of targeted interventions.

## Data Availability

The datasets generated and/or analyzed during the current study are not publicly available because they contain potentially sensitive participant-level survey responses. De-identified data may be made available from the corresponding author on reasonable request and subject to ethical and institutional considerations.
