# Supplementary material for: Knowledge, attitudes, and practices regarding vitamin D among adults in Palestine: a cross-sectional study
Source: Front Public Health. 2026 Jul 14;14:1868025. doi: 10.3389/fpubh.2026.1868025 (PMC13407759; doi:10.3389/fpubh.2026.1868025)
Supplement: Supplementary file 1 [file Data_Sheet_1.docx]

Questionnaire questions in English:

Demographic Information

1. Age:
- 18–25
- 26–35
- 36–45
- 46–60
- >60

2. Gender:
- Male
- Female

3. Place of residence:
- West Bank
- Gaza Strip
- Jerusalem
- Israel
- Palestinian living outside Palestine

4. Living area:
- City
- Village
- Camp

5. Governorate:
- Ramallah & Al-Bireh
- Hebron
- Nablus
- Bethlehem
- Jenin
- Tubas
- Tulkarm
- Qalqilya
- Salfit
- Jericho
- Jerusalem
- Other

6. Educational level:
- Primary
- Secondary (Tawjihi)
- Diploma / Vocational
- Bachelor’s degree
- Postgraduate (Master’s / PhD)

7. Employment status:
- Student
- Self-employed / Freelance work
- Employee (government/private)
- Not currently working
- Retired

8. Are you a healthcare worker/student?
- Yes
- No

9. If yes, specify specialty:
- Medicine
- Dentistry
- Pharmacy
- Nursing
- Medical Laboratory
- Other

10. Monthly income:
- Prefer not to answer
- <2000 NIS
- 2000–5000 NIS
- >5000 NIS
- Not working / Student

11. Marital status:
- Single
- Married
- Divorced
- Widowed

12. Do you currently smoke?
- Yes
- No

13. Do you have any chronic diseases?
- Yes
- No
If yes, specify:

Knowledge about Vitamin D
yes/no/I don’t know
- Vitamin D is important for bone health
- Vitamin D deficiency may cause bone pain
- Vitamin D deficiency may lead to osteoporosis
- Vitamin D is important for calcium absorption
- Vitamin D deficiency is a common cause of fatigue
- Vitamin D deficiency affects memory and concentration
- Vitamin D deficiency causes hair loss
- Vitamin D deficiency is a common cause of depression symptoms
- Vitamin D deficiency is a common cause of weak/low immunity
- Vitamin D supplements can help prevent bacterial and viral infections (eg. common cold, and influenza)
- Sunlight helps the body produce vitamin D
- Vitamin D can be obtained from food
- Fish and eggs contain vitamin D
- Vegetables are a main source of vitamin D

- Most people can get enough vitamin D from diet alone

- You can measure vitamin D levels by doing a blood test

(Response options for above: Yes / No / I don’t know)

Attitudes (Likert scale (1 to 5) : Strongly disagree (1) → Strongly agree (5))

- Vitamin D deficiency is common in our society
- Vitamin D deficiency may cause serious health problems
- I believe I am at risk of vitamin D deficiency
- Regular sun exposure is necessary for good health
- Sun exposure is more harmful than beneficial
- Vitamin D supplements should only be taken after testing
- Taking vitamin D improves health even without deficiency
- Vitamin D supplements are generally safe to be taken without medical advice/consultation
- I rely on social media as a primary source of health information
- I believe that regular vitamin D testing is necessary

Practices

- Which body parts are usually exposed to sunlight?
 Face only / Face & hands / Face, hands & arms / Larger body areas

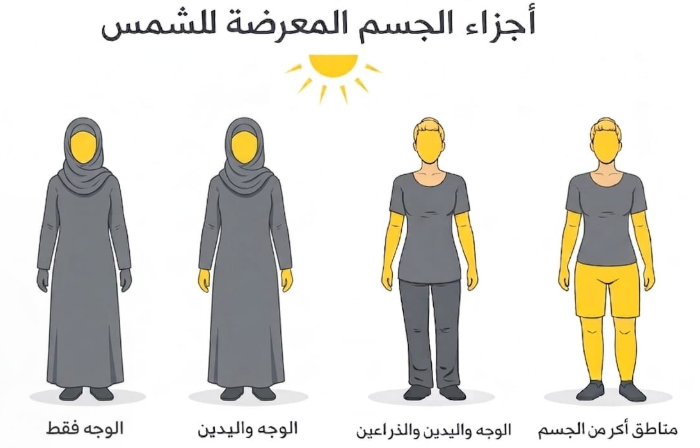

- How often are you exposed to sunlight?
 Daily / 3–4 times weekly / 1–2 times weekly / Rarely / Never

- Duration of daily exposure to sunlight:
 <10 min / 10–30 min / >30 min

- At what time do you usually get exposed to the sun (more than one answer applicable):
 Morning / Noon (10 AM–3 PM) / Evening (after 3 PM)

- Do you try to intentionally increase sun exposure?
 Yes / No

- If you develop symptoms (like fatigue, pain, hair loss), what do you do?
 Visit doctor / Take supplements / ignore the symptoms / Other

- Have you taken vitamin D supplements in the last 12 months?
 Yes, regularly / Yes, occasionally / No

Optional:
- Did you test vitamin D before starting? Yes / no
- When was your last test? In the last 12 months / more than 12 months ago / have never tested
- Do you know your dose of vitamin D? yes/ no
- Do you take high doses of vitamin D (eg. weekly/monthly regimens)? Yes/no
- Who recommended the supplement?
 Physician / Pharmacist / Self / Family/Friends / Social media / Other
- Why do you take vitamin D? (multiple answers)
 Diagnosed deficiency by blood test / Medical advice without testing/ Fatigue / Bone pain / Hair loss / To improve immunity / General health / Other
